# Controlled hypothermia may improve surfactant function in asphyxiated neonates with or without meconium aspiration syndrome

**DOI:** 10.1371/journal.pone.0192295

**Published:** 2018-02-08

**Authors:** Chiara Autilio, Mercedes Echaide, Daniele De Luca, Jesús Pérez-Gil

**Affiliations:** 1 Department of Biochemistry, Faculty of Biology and Research Institute Hospital 12 de Octubre, Complutense University, Madrid, Spain; 2 Laboratory of Clinical Molecular Biology, Department of Laboratory Medicine, “A. Gemelli” University Hospital, Catholic University of the Sacred Heart, Rome, Italy; 3 Division of Pediatrics and Neonatal Critical Care, “A. Béclère” Medical Center, South Paris University Hospitals, APHP, Paris, France; Science and Technology Facilities Council, UNITED KINGDOM

## Abstract

**Background:**

Whole-body hypothermia (WBH) is used to improve neurological outcomes in perinatal asphyxia. Recent studies suggested a beneficial effect of hypothermia for some types of acute respiratory failure. However, no data are available about the biophysical function of human surfactant during WBH. We investigated whether WBH improves surfactant biophysical properties in asphyxiated neonates with or without meconium aspiration syndrome (MAS).

**Methods:**

Non-bronchoscopic bronchoalveolar lavage (BAL) has been collected from 10 asphyxiated neonates (2 with MAS, 8 with no lung disease (NLD)) at different time-points (pre-WBH, 24h, 48h, 72h of WBH and post-WBH). Surfactant was extracted and tested by captive bubble surfactometry (CBS) in triplicate, at 37°C and 33.5°C, through initial adsorption and dynamic compression-expansion cycling. Phosphatidylcholine and cholesterol were assayed using enzymatic methods. Clinical data were recorded in real-time.

**Results:**

Minimum surface tension under dynamic testing was significantly improved as assessed at 33.5°C compared with its behavior at 37°C in NLD neonates: the difference was evident after at least 72h of WBH and remained significant at 6h after rewarming (72h: *p* = 0.009; rewarming: *p* = 0.040). Similar results were obtained in MAS patients whose surfactant activity improved already at 48h of hypothermia. Total cholesterol showed a trend to increase at the first 24-48h of hypothermia in NLD patients. Conversely, hypothermia seemed to reduce the excess of exogenous cholesterol in MAS surfactant.

**Conclusions:**

Surfactant biophysical properties may improve after 48-72h of WBH in asphyxiated neonates and the improvement is maintained shortly after rewarming. Due to study limitations, further studies are warranted to better clarify the effects of hypothermia on surfactant activity.

## Introduction

Controlled hypothermia is an effective treatment for encephalopathy due to perinatal asphyxia. Cooling is usually applied as whole-body hypothermia (WBH) at a constant servo-controlled temperature of 33.5°C, starting early from birth and continuing for the following 72h [1–3]. The beneficial effect of WBH on the neonatal brain is well known but there are scanty data about its effect on other organs. Short preliminary reports have suggested a possible usefulness of hypothermia for acute respiratory distress syndrome (ARDS) or necrotising enterocolitis [4–7]. In the newborn lung, this effect might be due to the reduction in inflammatory mediators and to the improvement of lung mechanics [8,9]. Thus, controlled hypothermia could be theoretically useful for respiratory conditions characterised by high lung tissue inflammation. For instance, WBH seems to improve short term oxygenation and clinical outcomes in neonates with meconium aspiration syndrome (MAS), which is often associated with perinatal asphyxia [10].

However, the effect of WBH on the lung might be more complex, as it could theoretically affect surfactant function, as well. In fact, according to the scanty data available on this topic: 1) WBH seems to change surfactant adsorption overtime and reduce lung inflammation [8,9]; 2) *in vivo* metabolic replacement of disaturated-phosphatidylcholine (DPPC) does not change during WBH [11]; 3) animal and *in vitro* studies show that hypothermia may lead to changes in surfactant phospholipid composition [12–14]. These changes may be multifaceted and generate complex interactions. For instance, anionic phosphatidylglycerol and cholesterol are ≈10% of total surfactant lipids [15] but all phospholipids contribute for the progressive reduction of the surface tension at the interface, achieving an equilibrium value of around 20 mN/m [15]. This process, called surfactant initial adsorption, seems to be improved by an increase in fluidity: cholesterol, as is the case for unsaturated phospholipids, seems to enhance the adsorption of surfactant at both 37°C [16] and 25° C [17]. However, fluidity of surfactant membranes alone does not allow reaching low surface tensions during expiration [18]. Conversely, DPPC is able to generate extremely high packing levels under compression, reaching surface tension values close to 0 mN/m [19]. However, and according to the lipid composition, surfactant films show different compression-driven phase transitions at temperature in the physiological range [16, 20].

Beyond these scanty data, to the best of our knowledge, no specific studies have investigated the biophysical function of human surfactant during controlled hypothermia. This lack of data is due to the difficulty in obtaining newborn samples, the low concentration of recovered material and the complexity of available techniques for testing surfactant activity [15]. Up to now, only a case report showed an improvement in surfactant function during WBH of a baby whose amniotic fluid was meconium stained [21].

Thus, our aim was to perform a biophysical study on surfactant properties by using non-bronchoscopic broncho-alveolar lavages (BAL) from neonates under WBH. We hypothesized that WBH might improve surfactant activity. To address this hypothesis, we tested BAL samples before, during and after WBH by captive bubble surfactometry (CBS).

## Material and methods

### Subjects

Eligible babies were asphyxiated neonates admitted to our neonatal intensive care unit (NICU) during 2015 and requiring WBH. Exclusion criteria were congenital lung disease, complex malformations or blood-staining of BAL samples. WBH has been provided following TOBY trial criteria [22], using whole body, core temperature servo-controlled, water-filled mattresses set at 33.5°C. In our NICU, following our routine care protocol, asphyxiated neonates needing WBH are kept intubated and ventilated until complete rewarming (at 37°C), even though they do not need that for pulmonary reason [9], in order to reduce the metabolic demand. Extubation always takes place 6h after rewarming.

Neonates with no lung disease (NLD) were ventilated using time-cycled, pressure-regulated, assisted-controlled ventilation: our targets were 4–6 mL/kg tidal volume, pH between 7.4 and 7.20 and PaCO_2_ between 35 and 65 mmHg; positive end expiratory pressure was 4-6cmH_2_O and FiO_2_ was set as low as possible to keep saturation between 90% and 95% and PaO_2_ between 50 and 70 mmHg; NLD babies were switched to synchronised intermittent mandatory ventilation to avoid hypocapnia, when necessary.

Neonates with MAS were treated with high frequency oscillatory ventilation (HFOV), as conventional ventilation needed peak pressure > 22cmH_2_O or a tidal volume > 6 ml/kg to reach the above described targets. HFOV was started within the first hour of life at a mean airway pressure (Paw) of 2 cmH_2_O higher than Paw provided in conventional ventilation; optimum lung volume strategy was applied and alveolar recruitment was performed, as needed [23]. Frequency was 12–9 Hz and oscillation amplitude was adjusted to obtain visible chest oscillation; both frequency and amplitude were titrated according to blood gas values and targeting to deliver volume of ≤2.5 mL/kg [24]. Sedation was provided with remifentanyl infusion at 0.1–2 mcg/kg/min. Neonates were continuously monitored with amplitude-integrated EEG and anticonvulsant drugs were used as necessary. All patients had an indwelling arterial line and arterial blood gases values were used to evaluate the respiratory status and calculate the oxygenation index (OI = FiO_2_ x mean airway pressure/PaO_2_) and the PaO_2_/FiO_2_ ratio. Basic clinical data were real time registered in our electronic NICU database. The study protocol was approved by the local ethical committee of the South Paris University Hospitals (n.PP13-046) and parental consent was obtained upon NICU admission.

**Fig 1:**
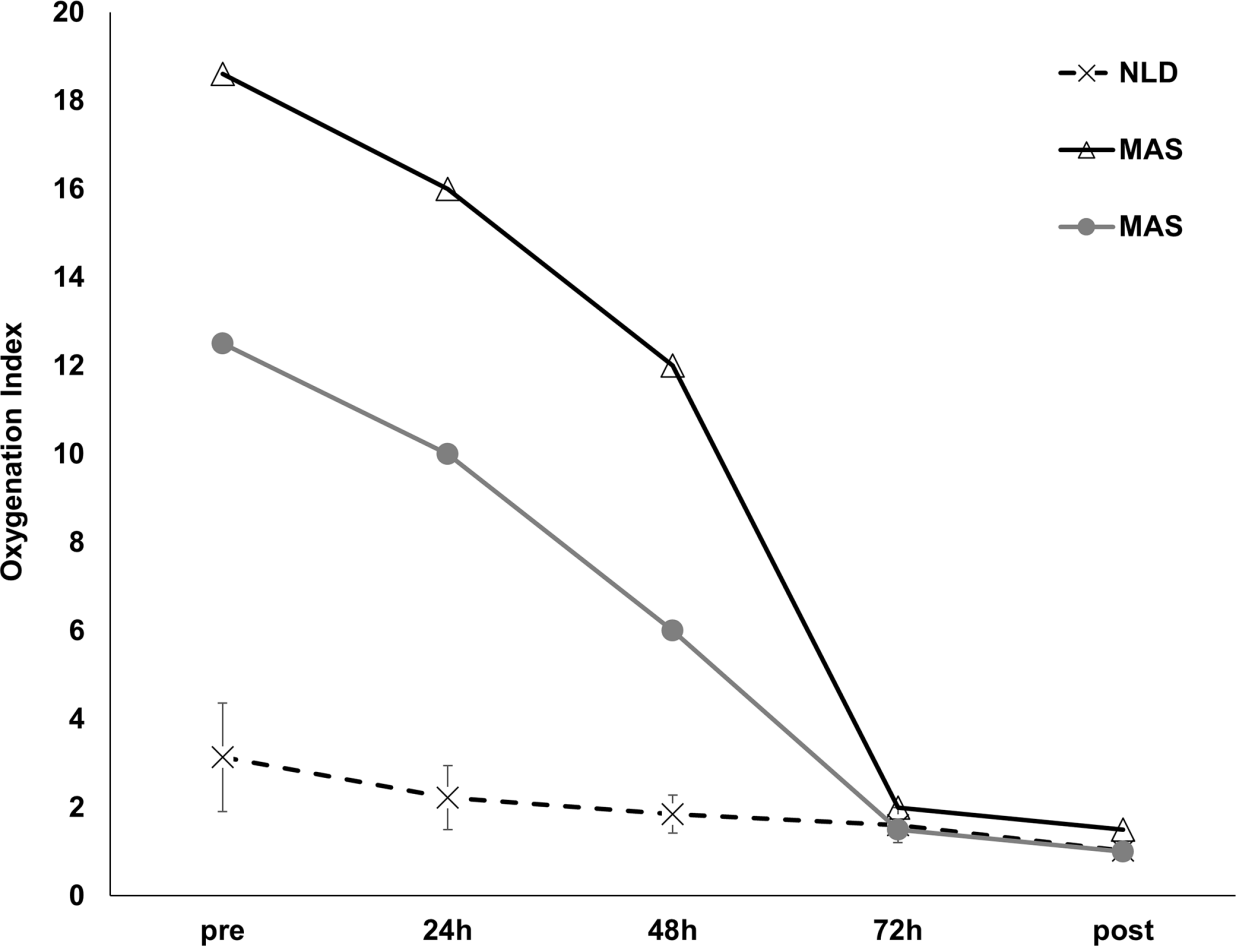
Oxygenation index (OI) trend in enrolled patients. Hatched line represents mean OI in neonates with no lung disease (NLD) and T-bars represent their standard deviation. Open triangles and full grey circles represent the two neonates with meconium aspiration syndrome (MAS). Abbreviations: MAS: meconium aspiration syndrome; NLD: no lung disease; OI: oxygenation index.

Fifteen patients were eligible; four were excluded because of blood-staining and one because he died at 50h of life (that is, before completing WBH treatment) from multi-organ failure secondary to perinatal asphyxia. Ten patients were finally enrolled and their characteristics are detailed in Table 1. All patients survived and were successfully discharged. Eight patients did not show any lung disease (NLD group). They had normal amniotic fluid and no signs of infection; chest imaging and clinical examination were always normal and they never needed any supplemental oxygen. Two patients were diagnosed with meconium aspiration (MAS group), as they had meconium-stained amniotic fluid and secretions upon tracheal suctioning; they developed respiratory distress early from birth and had chest X-rays or lung ultrasound [25] typical for MAS. Fig 1 illustrates the OI for NLD babies and the two patients affected by MAS. As per our internal protocol, these patients received broncho-alveolar lavage [26] with diluted poractant-α (Curosurf^®^, Chiesi Farmaceutici, Parma, Italy; 75 mg/kg in 15 mL/kg 0.9% saline), immediately followed by 200 mg/kg poractant-α within the first 24h of life. For these patients the first BAL was performed before the onset of WBH and before any surfactant therapy.

**Table 1:** Basic characteristics of all enrolled neonates. Data are expressed as mean (standard deviation) or numbers (%). NLD neonates were intubated for delivery room resuscitation and kept ventilated to reduce their metabolic demand during hypothermia, according to our routine clinical protocol. All patients were discharged home with no respiratory support and with normal neurological exam and brain magnetic resonance imaging.

Neonates	10
Gestational age (weeks)	39 (1.5)
Birth weight (grams)	3138 (251)
SNAPPE-II score	52 (10.5)
5’ Apgar score	5 (1.9)
Caesarean section	7 (70%)
Sex	4 females/6 males
OI at the NICU admission	3 (1.9)
Diagnosis	8 NLD/2 MAS

**Abbreviations**: MAS: meconium aspiration syndrome; NLD: no lung disease; OI: Oxygenation index; NICU: Neonatal intensive care unit; SNAPPE-II Score for Neonatal Acute Physiology-Perinatal Extension-II.

### Protocol for sampling

According to our clinical care protocol, patients received a non-bronchoscopic BAL before the onset of WBH and then every 24h to perform microbiological surveillance or secretions removal. The last BAL was performed just before extubation. BALs were done only when neonates needed to be suctioned for clinical reasons: no procedure was performed solely for the study purposes and no change was provided to the routine clinical assistance. The first BAL was considered as control measurement under normothermia for the study purpose.

Lavages were performed with a well-standardised procedure already described elsewhere [27] and according to the European Respiratory Society advices [28]. In detail, the neonate was placed supine with the head turned to the left so that the right lung would be predominantly sampled. One mL/kg 0.9% NaCl (at the patients’ temperature) was instilled through an end-hole catheter of suitable diameter, inserted into the endotracheal tube through a Y-piece, while continuing ventilation and until a resistance was felt. After three ventilator cycles, the suction was gently applied and the fluid was aspirated into a sterile polypropilene trap with 50 mmHg of negative pressure. This procedure was repeated with the head turned to the right, so that the left lung would be predominantly sampled and the two aliquots recovered were pooled. No sample was visibly blood-stained. On average, 46% of the instilled volume was recovered, which means ≈1mL for each sample [27]. BALs were performed by trained nurses under the attending physicians’ supervision. Samples were immediately divided into two aliquots: 0.3 mL were sent for microbiological culture, while the remaining volume was centrifuged (700*g*;10’;4°C) to remove the pellet; the supernatant was immediately frozen at -35°C.

### Measurements

**Fig 2:**
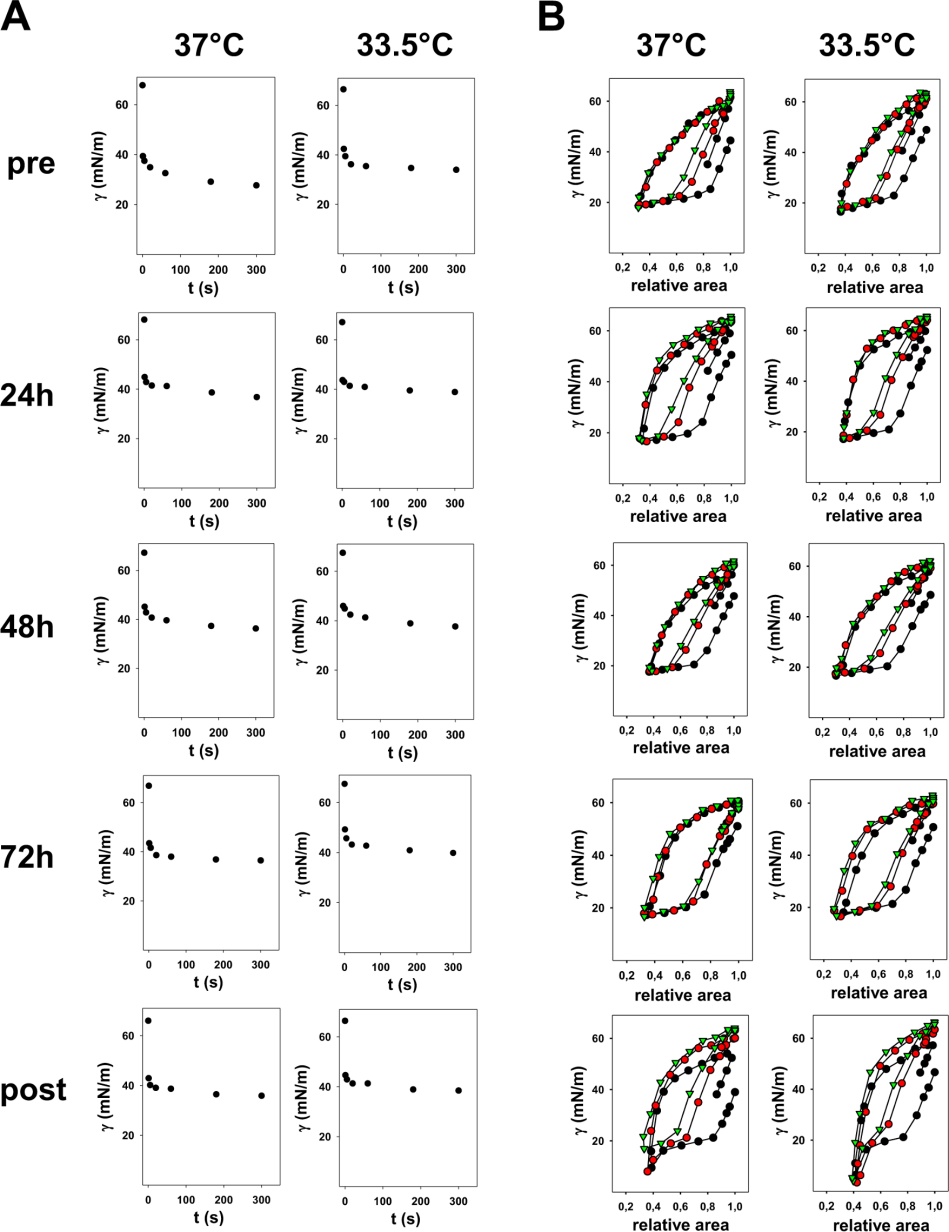
Illustrative case of a patient studied through a complete CBS experiment. Surfactant biophysical properties were studied both under initial adsorption (A) and compression-expansion dynamic cycling (B), mimicking the spontaneous respiratory cycles, at two temperatures (37°C and 33.5°C). Only one of three different replicates of the whole experiment is shown. Acronyms indicate samples obtained before (pre), during (24h, 48h, 72h) and after (post) WBH. Dynamic cycles n°1, 10, and 20 are depicted by black and red circles and green triangles, respectively. This patient had perinatal asphyxia encephalopathy but was not affected by MAS. Abbreviations: γ: surface tension (in mN/m); CBS: captive bubble surfactometry; MAS: meconium aspiration syndrome; WBH: whole body hypothermia.

To precipitate surfactant, BAL supernatant was ultracentrifuged (100000*g*; 4°C;1h), and diluted with 5 mM Tris buffer, containing 150 mM NaCl (5mM; pH7) (Sigma-Aldrich, Germany), to a PC concentration of 8 mg/mL. We did not pool the material of different patients to take the biological variations into account. PC and cholesterol were assayed in triplicate using previously published enzymatic methods (Spinreact, St. Esteve de Bas, Girona, Spain) [29,30]. After surfactant precipitation, we obtained 50 μg of PC for each sample. Biophysical activity was evaluated applying 150 nL of surfactant suspension (8 mg/mL) in CBS at a frequency of 20 cycles/min. CBS mimics the cyclic changes in alveolar volume during breathing cycles [31]. The study of surface-active properties under dynamic compression-expansion conditions is performed at the air-liquid interface of an air bubble inside a buffer-filled chamber. The size of the bubble is compressed and expanded periodically due to the hydrostatic pressure delivered by a piston. Resulting changes in the bubble shape are continuously recorded and analysed, permitting the calculation of volume, area, and surface tension at any time [32]. The chamber was filled with a buffer solution of 5 mM Tris and 150 mM NaCl, pH 7, containing 5% sucrose (Sigma-Aldrich, St.Louis, MI, USA) to increase density and allow surfactant to float against the bubble surface. This mimics the capillary layer of surfactant coating the alveolar surface. For each sample, CBS measurements were carried out in triplicates, both at 33.5°C and at 37°C. The changes in bubble shape were monitored for 5’ and related to changes in surface tension (γ). The chamber was then sealed to perform dynamic cycling in which the bubble was continuously expanded and compressed for 20 cycles and its changes in area and surface tension measured overtime. We also evaluated the compressibility, as the capability of each surfactant sample to reduce surface tension under minimal variations in bubble compression. This is done calculating the slope dimensionless value of the line passing through the minimum and maximum points of compression in the dynamic cycling curve (minimum and maximum area of the bubble) during the last cycle. The steeper is the slope, the lower is surfactant compressibility and the work needed to achieve minimal surface tension. The Fig 2 shows CBS results of an illustrative case: the complete CBS analysis required a mean of 10 (1.5) days per each patient.

### Data analysis

Data are expressed as mean (standard deviation) and analysed with repeated-measures ANOVA: within-subject contrasts for the different time-points (pre-WBH, 24h, 48h, 72h of WBH and post-WBH) have been performed. In detail, data about the initial surfactant adsorption have been analysed with multivariate repeated-measures ANOVA, considering the time-points within the experiment (measurements at 0”, 1”, 5”, 20”, 60”, 180” and 300”) and the time-points of hypothermia treatment (pre, 24h, 48h, 72h of WBH and post) as covariates. Paired comparisons between data obtained from same samples performing experiments at different temperatures (33.5 and 37°C) have been performed with paired Student *t*-test. Comparison between neonates with or without MAS have been performed with unpaired Student *t*-test. Correlation analysis has been performed using Pearson’s coefficient. Analyses have been performed with SPSS 15.0 (SPSS inc., Chicago, IL, USA) and *p*<0.05 has been considered as significant.

## Results

**Fig 3:**
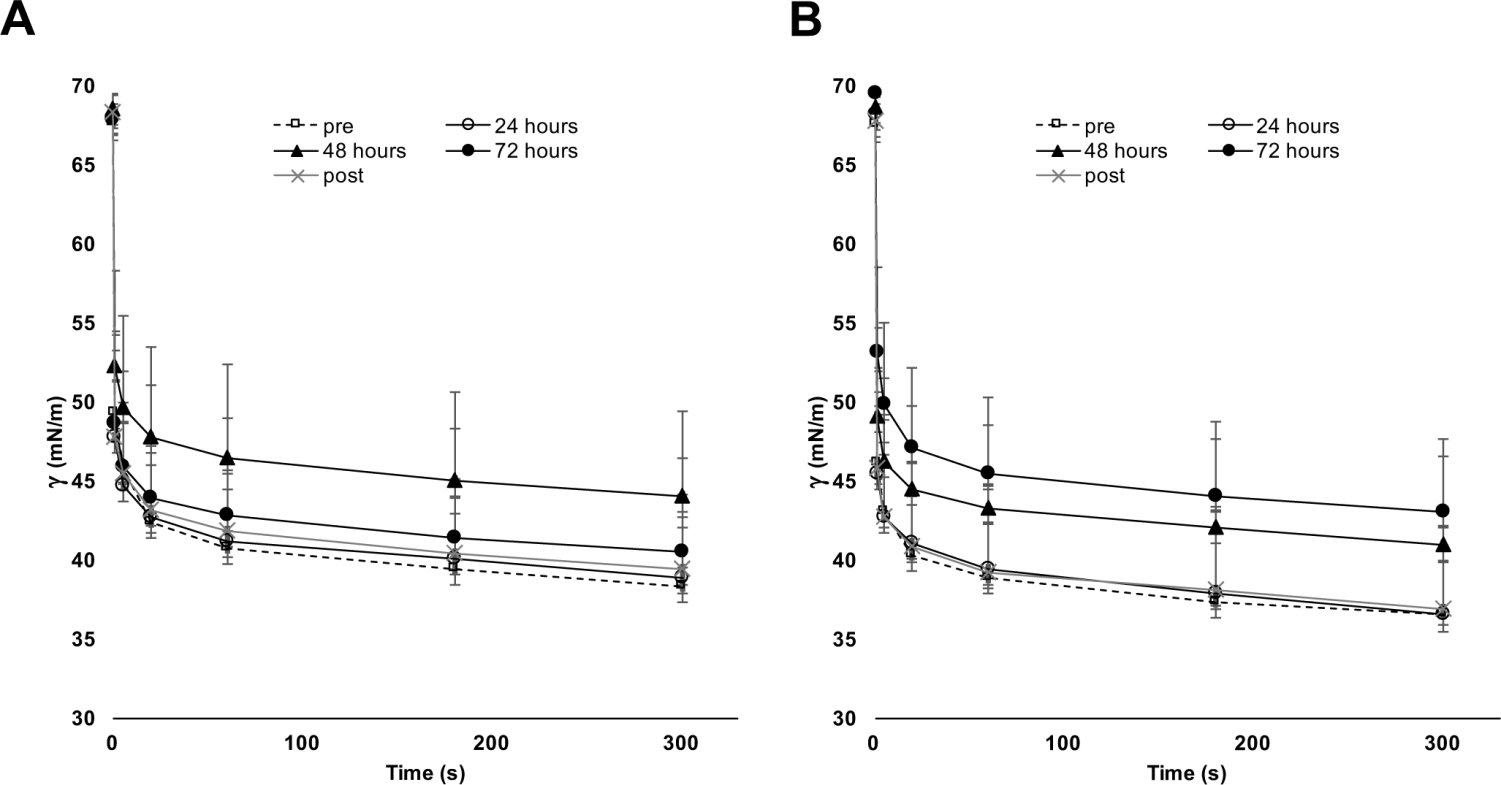
Initial adsorption. Adsorption is measured in terms of surface tension (γ) before and after the injection of surfactant for the following 5’. Panel A and B show experiments at 37°C and 33.5°C, respectively. Different symbols indicate samples obtained before (pre), during (24h, 48h, 72h) and after (post) WBH. T-bars represent the standard deviation of measurements obtained in 10 patients (8 NLD and 2 MAS). **Abbreviations**: γ: surface tension (in mN/m); MAS: meconium aspiration syndrome; WBH: whole body hypothermia.

Fig 3 shows that no significant differences are evident in surfactant initial adsorption at different time-points within the patients, irrespective of the experimental temperature (*p* = 0.484 and *p* = 0.281, for 37°C and 33.5°C, respectively). During the first 20” of adsorption, there is a quick surface tension decay both at 37°C and 33.5°C, and then a further slow decrease towards the apparent equilibrium (see Fig 3 and Table 2). While reaching the equilibrium, initial adsorption seems slightly worse for the experiment at 33.5°C, than for that at 37°C. Data were significantly different for the fast kinetics (that is, for 1” and 5” of initial adsorption) for the whole cohort [33.5°C (at 1”: 49.2 (5.1) mN/m; at 5”: 46.3 (4.9) mN/m) and 37°C (at 1”: 46.8 (5.1) mN/m, overall *p* = 0.047; at 5” 43.9 (4.9) mN/m, overall *p* = 0.046] and for NLD subgroup [33.5°C (at 1”: 48.4 (5.1) mN/m; at 5”: 46.0 (5.0) mN/m) and 37°C (at 1”: 46.2 (5.2) mN/m, overall *p* = 0.023; at 5” 43.4 (5.1) mN/m, overall *p* = 0.047]. All data and *post-hoc* comparisons are shown in Table 2. Once the equilibrium has been reached (that is, beyond the first 20”) no differences are evident between the two temperatures.

**Table 2:** Initial adsorption. Surface tension (γ_min_) in mN/m achieved during each time point of the initial adsorption from the injection of surfactant (t = 0 s) to the end (t = 300 s) of the experiment. Data are expressed as mean (standard deviation).

		**Whole cohort**						
**T (°C)**	**samples**	**t (s)**						
** **	** **	**0**	1 (*)	5 (**)	**20**	**60**	**180**	**300**
**37**	**pre**	67.5 (1.4)	46.1 (5.9)	43.1 (6.1)	40.3 (5.9)	38.9 (5.9)	37.4 (5.8)	36.5 (5.5)
**33.5**		67.6 (0.9)	49.3 (5.1)	46.0 (4.2)	42.4 (4.8)	40.7 (5.0)	39.4 (4.5)	38.3 (4.4)
**37**	**24h**	68.2 (1.1)	45.5 (4.2)	42.8 (4.7)	41.1 (5.1)	39.5 (5.3)	37.9 (5.5)	36.5 (5.6)
**33.5**		68.0 (0.6)	47.8 (6.5)	44.7 (7.3)	42.8 (8.3)	41.2 (7.8)	40.1 (8.2)	38.9 (7.6)
**37**	**48h**	68.7 (1.0)	49.1 (5.6)	46.2 (5.2)	44.5 (5.3)	43.3 (5.3)	42.0 (5.6)	41.0 (5.6)
**33.5**		68.5 (0.9)	52.2 (6.1)	49.6 (5.8)	47.7 (5.7)	46.5 (5.9)	45.1 (5.5)	44.1 (5.4)
**37**	**72h**	68.0 (1.3)	45.7 (5.3)	43.6 (5.1)	41.5 (4.9)	40.4 (4.7)	38.9 (4.7)	38.2 (4.6)
**33.5**		69.5 (1.3)	48.6 (4.6)	45.9 (4.1)	44.0 (3.7)	42.8 (3.6)	41.4 (3.7)	40.5 (3.6)
**37**	**post**	67.6 (0.8)	46.1 (4.9)	43.9 (3.9)	41.7 (3.2)	40.2 (3.1)	38.9 (3.0)	37.8 (2.9)
**33.5**		68.3 (1.2)	47.8 (3.6)	45.5 (3.2)	43.2 (2.1)	41.9 (2.6)	40.4 (2.6)	39.5 (2.6)
		**NLD**						
**T (°C)**	**samples**	**t (s)**						
** **	** **	**0**	**1 (#)**	**5 (##)**	**20**	**60**	**180**	**300**
**37**	**pre**	67.4 (1.6)	45.8 (6.9)	43.1 (7.2)	40.6 (6.5)	39.1 (6.4)	37.4 (6.4)	36.5 (6.1)
**33.5**		67.5 (1.0)	48.4 (5.2)	45.6 (4.9)	42.9 (4.7)	41.5 (4.5)	40.0 (3.7)	38.9 (3.8)
**37**	**24h**	68.2 (1.2)	45.9 (4.5)	43.0 (5.1)	41.5 (5.5)	40.1 (5.6)	38.6 (5.7)	37.4 (5.6)
**33.5**		67.8 (0.3)	48.3 (7.1)	45.5 (7.9)	43.4 (9.1)	41.9 (8.5)	40.8 (9.0)	39.7 (8.2)
**37**	**48h**	68.5 (1.1)	48.8 (6.5)	45.7 (6.0)	43.9 (6.1)	42.6 (6.0)	41.2 (6.3)	40.0 (6.2)
**33.5**		68.4 (0.9)	51.8 (7.3)	49.2 (7.0)	47.4 (6.9)	46.2 (7.1)	44.6 (6.7)	43.7 (6.5)
**37**	**72h**	67.8 (1.3)	44.5 (5.2)	42.5 (5.3)	40.4 (5.2)	39.3 (5.1)	38.1 (5.1)	37.5 (5.0)
**33.5**		67.6 (0.7)	47.4 (1.8)	44.9 (2.4)	43.2 (2.6)	42.1 (2.9)	40.8 (3.3)	40.0 (3.1)
**37**	**post**	67.7 (0.8)	45.8 (4.9)	42.8 (3.9)	40.8 (3.2)	39.2 (3.1)	38.1 (3.0)	36.9 (2.9)
**33.5**		68.2 (1.2)	46.9 (2.6)	44.7 (2.4)	42.5 (2.3)	41.3 (2.3)	39.8 (2.2)	38.9 (2.2)

Post hoc comparisons between the two temperatures for the fast kinetics period (that is, 1” and 5” of initial adsorption)

(*) Whole cohort p values at 1”: pre (0.040), 24h (0.033), 48h (0.016), 72h (0.053) and post (0.005)

(**) Whole cohort p values at 5”: pre (0.010), 24h (0.045), 48h (0.002), 72h (0.024) and post (0.011)

(#) NLD group p values at 1”: pre (0.022), 24h (0.071), 48h (0.030), 72h (0.045) and post (0.015): p = 0.047

(##) NLD group p values at 5”: (0.008), 24h (0.022), 48h (0.004), 72h (0.017) and post (0.049).

**Fig 4:**
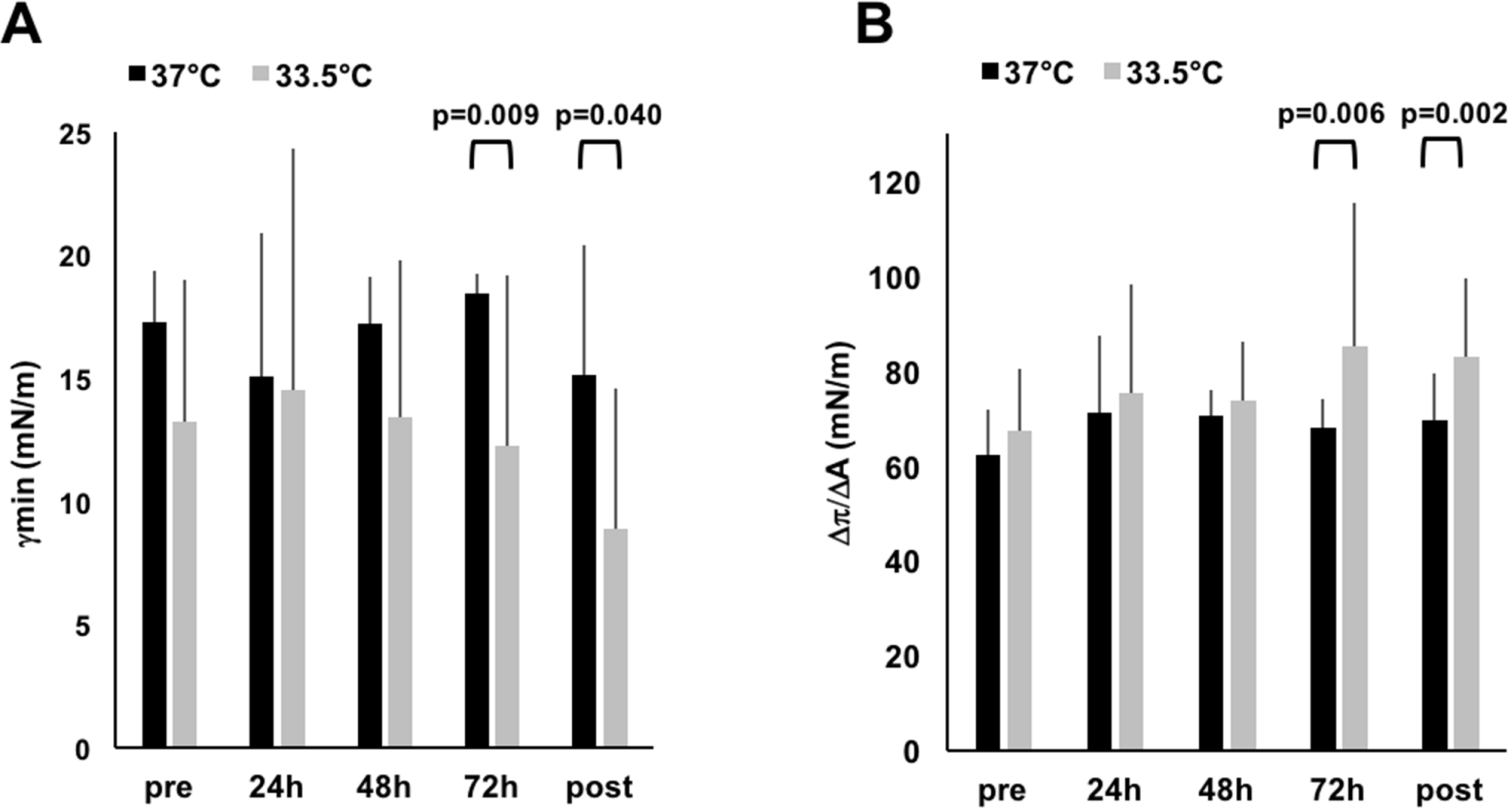
(A) Dynamic compression-expansion cycling. Black and grey columns represent the minimum surface tension (γ_min_) expressed as mN/m during the whole dynamic experiments at 37°C and 33.5°C, respectively. T-bars represent the standard deviation of measurements obtained in 8 NLD patients. X-axis indicates timepoints: before (pre), during (24h, 48h, 72h) and after (post) WBH. No significant differences are evident over time during WBH within the same patients, neither testing at 37°C, nor at 33.5°C. The black arches indicate the significant differences at the paired comparison between data obtained at the two different temperatures. The respective *p*-value is also showed. γ_min_ at the two temperatures is not significantly different before WBH, at 24h and 48h of hypothermia. (B) Compressibility. Black and grey columns represent the slope value (ΔΠ/ΔA) expressed as mN/m of the 20^th^ dynamic cycle obtained at 37°C and 33.5°C, respectively. The value was calculated as the slope of the line passing through the minimum and maximum points of the area generated by 20^th^ compression cycle. T-bars represent the standard deviation of measurements obtained in 8 NLD patients. X-axis indicates timepoints: before (pre), during (24h, 48h, 72h) and after (post) WBH. No significant differences are evident over time during WBH within the same patients, neither testing at 37°C, nor at 33.5°C. The black arches indicate the significant differences at the paired comparison between data obtained at the two different temperatures. The respective *p*-value is also showed. ΔΠ/ΔA at the two temperatures is not significantly different before WBH, at 24h and 48h of hypothermia. Abbreviations: γ: minimum surface tension (in mN/m); MAS: meconium aspiration syndrome; WBH: whole body hypothermia; ΔΠ/ΔA: slope value.

Fig 4A shows that minimum surface tension achieved during all dynamic cycles does not change at different time-points within NLD patients, neither when the experiments were performed at 37°C (*p* = 0.788), nor at 33.5°C (*p* = 0.649). There is no significant difference in minimum surface tension at each time-point between NLD and MAS neonates when measurements are performed at 37°C (pre: *p* = 0.663; 24h: *p* = 0.99; 48h: *p* = 0.99; 72h: *p* = 0.466; post: *p* = 0.99) or 33.5°C (pre: *p* = 0.744; 24h: *p* = 0.537; 48h: *p* = 0.551; 72h: *p* = 0.409; post: *p* = 0.884). When running paired comparisons at each time-points between experiments performed at different temperatures, surface tension obtained at 33.5°C (pre: 13.3 (5.8) mN/m; 24h: 14.5 (9.8) mN/m; 48h: 13.4 (6.4) mN/m; 72h: 12.3 (6.9) mN/m; post: 8.9 (5.7) mN/m) was significantly lower than that obtained at 37°C (pre: 17.3 (2.1) mN/m; 24h: 15.1 (5.8) mN/m; 48h: 17.2 (1.9) mN/m; 72h: 18.4 (0.8) mN/m; post: 15.1 (5.3) mN/m) from 72h of WBH onwards and the difference remains significant after rewarming (Fig 4A). At the same time, the compressibility value was significantly higher at 33.5°C (pre: 67.5 (13.0) mN/m; 24h: 75.4 (22.7) mN/m; 48h: 73.9 (12.3) mN/m; 72h: 85.0 (30.3) mN/m; post: 83.1 (16.5) mN/m) than at 37°C (pre: 62.4 (9.3) mN/m; 24h: 71.0 (16.3) mN/m; 48h: 70.6 (5.4) mN/m; 72h: 67.9 (6.2) mN/m; post: 69.4 (10.0) mN/m) from 72h of WBH onwards (Fig 4B).

**Fig 5:**
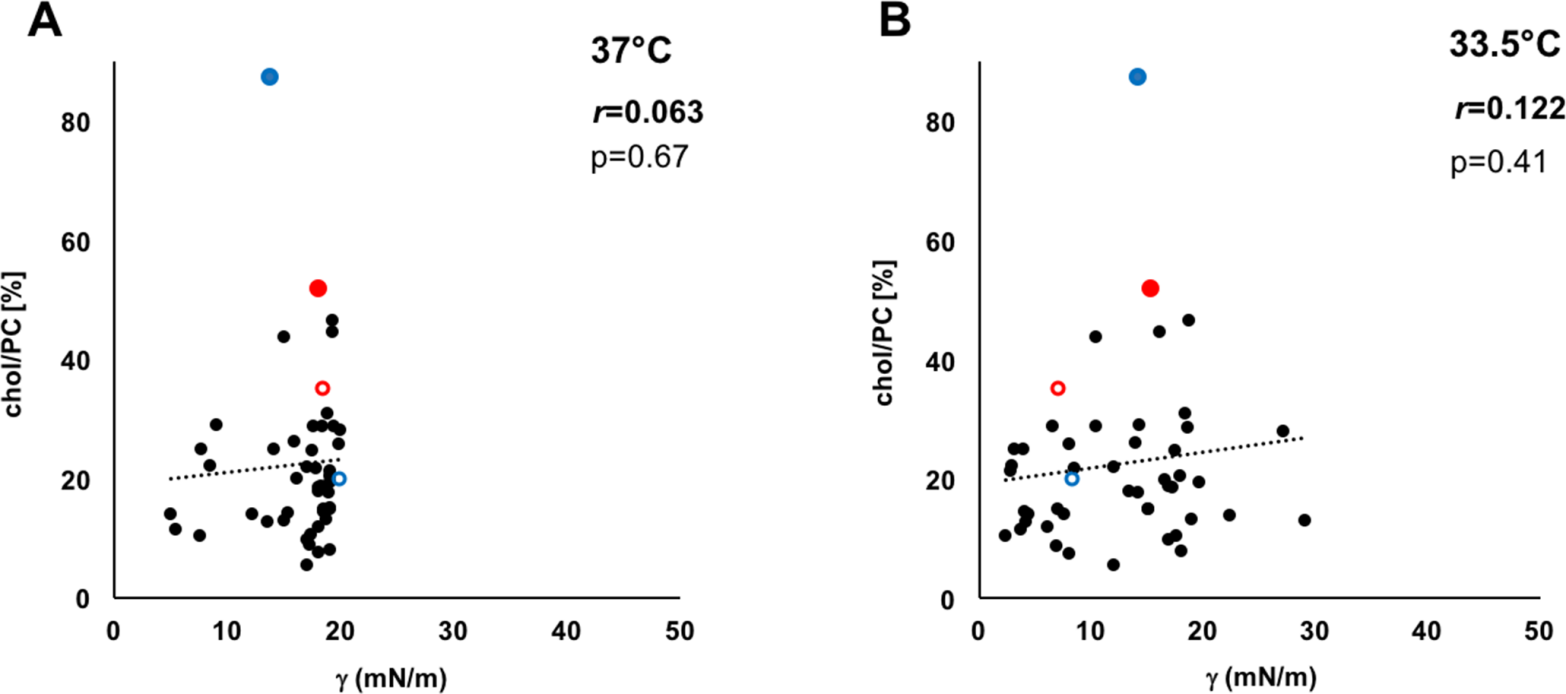
Correlation analysis. Correlation plots between cholesterol amount expressed as percentage with respect to PC choline (Chol/choline) and minimal surface tension (γ_min_) achieved during dynamic cycles at 37°C (A) and 33.5°C (B). The dotted line shows the trend line of values dispersion. Correlation coefficients and *p*-values are also showed. Red and blue circles indicate MAS patient n.1 and n.2, respectively (filled circle: pre-hypothermia, empty circle: post hypothermia). No significant correlations are evident at the two temperatures, considering the whole population. **Abbreviations**: γ: minimum surface tension (in mN/m); Chol/Choline: cholesterol amount (in % with respect to total PC-derived choline).

Table 3 shows minimal surface tension, compressibility and cholesterol amounts. Surface tension is significantly lower and compressibility is significantly higher at 33.5°C than at 37°C, both at 72h of WBH and post-hypothermia. Total cholesterol did not significantly change but seemed to show a tendency to slightly increase in 5 out of 8 patients at 24h and 48h of WBH before coming back to basal values at 72h (pre: 21.6 (5.7) %; 24h: 26.0 (11.5) %; 48h: 24.0 (11.9) %; 72h: 20.3 (11.5) %; post: 16.2 (7.6) %; *p* = 0.378). There were no significant correlations between cholesterol amount and minimal surface tension at both 37°C and 33.5°C, for all patients (Fig 5A and 5B).

**Table 3:** Minimal surface tension, compressibility and cholesterol amounts of samples tested before (pre), during (24h, 48h, 72h) and after (post) WBH at 33.5°C and 37°C. Data are expressed as mean (standard deviation).

T (°C)	samples	NLD		
		ϒ min (mN/m)	ΔΠ/ΔA (mN/m)	chol/PC choline (%)
**37**	**pre**	17.3 (2.1)	62.4 (9.3)	21.6 (5.7)
**33.5**		13.3 (5.8)	67.5 (13.0)	
**37**	**24h**	15.1 (5.8)	71.0 (16.3)	26 (11.5)
**33.5**		14.5 (9.8)	75.4 (22.7)	
**37**	**48h**	17.2 (1.9)	70.6 (5.4)	24.0 (11.9)
**33.5**		13.4 (6.4)	73.9 (12.3)	
**37**	**72h**	18.4 (0.8) *	67.9 (6.2) #	20.3 (11.5)
**33.5**		12.3 (6.9) *	85.0 (30.3) #	
**37**	**post**	15.1 (5.3) **	69.4 (10.0) ##	16.2 (7.6)
**33.5**		8.9 (5.7) **	83.1 (16.5) ##	

* p = 0.009

** p = 0.040

# p = 0.006

## p = 0.002.

**Fig 6:**
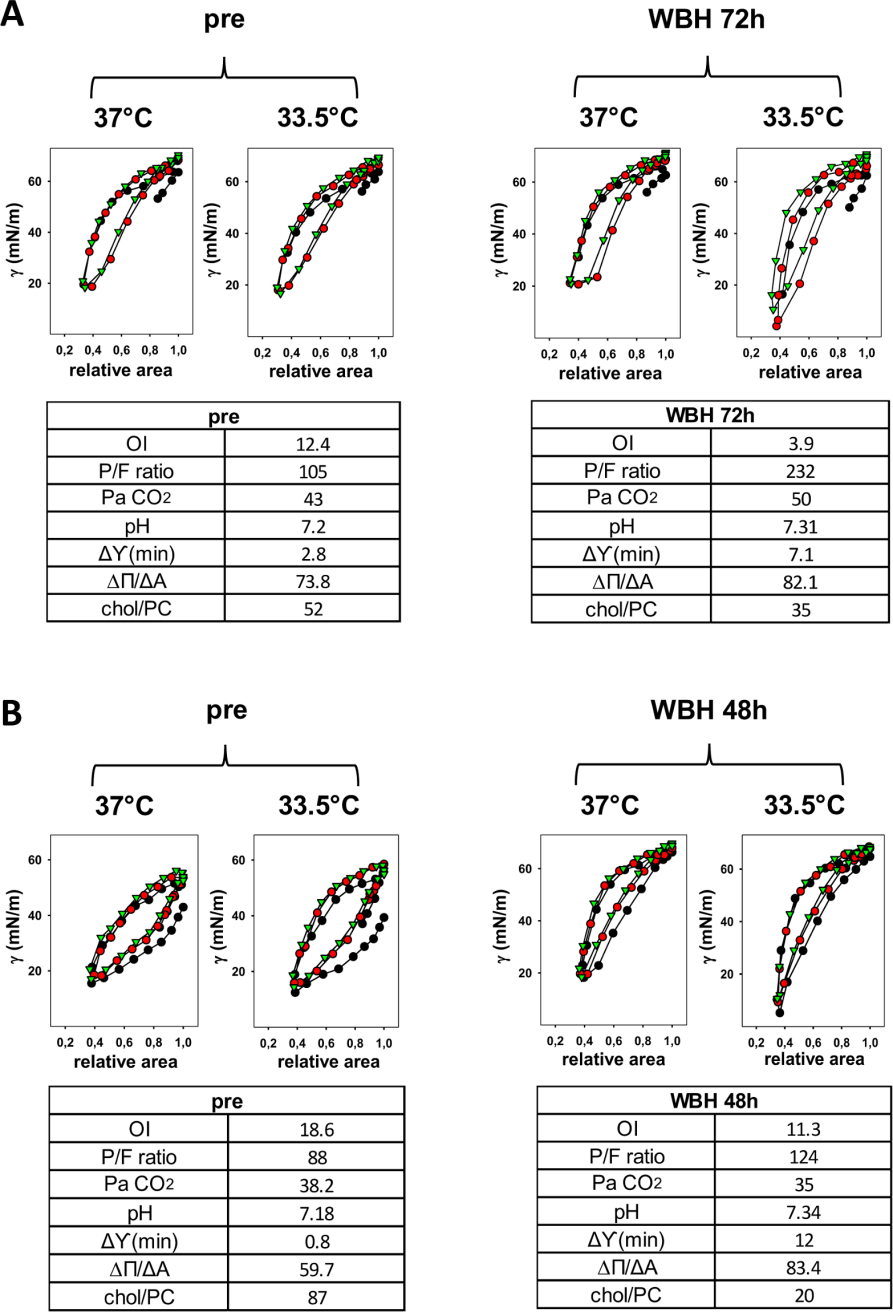
An illustrative dynamic compression-expansion cycling for the two patients with meconium aspiration syndrome before hypothermia and when the best surfactant performance is achieved during hypothermia. Panel A and B represent patient 1 and 2, respectively. BAL sampling time-points are indicated as (pre), (48h) and (72h). Experiments were performed at 37°C and 33.5°C. Cycles number 1, 10, and 20 are depicted by black and red circles and green triangles, respectively. Oxygenation, gas exchange data, Δ (minimum surface tension—γ_min_) during the whole dynamic experiments between measurements tested at 37°C and 33.5°C, compressibility at the 20^th^ cycle (ΔΠ/ΔA), chol/PC choline are shown for each time-point and each patient in the inserted tables. PaCO_2_ is expressed in mmHg, γ_min_ and ΔΠ/ΔA are expressed as mN/m, cholesterol is expressed as % of surfactant PC. OI, P/F ratio, pH are dimensionless numbers. **Abbreviations**: γ: surface tension (in mN/m); MAS: meconium aspiration syndrome; OI: oxygenation index; P/F ratio: PaO_2_/FiO_2_; WBH: whole body hypothermia.

MAS patients presented the basic characteristics described in Table 4: both were very critically ill and had pulmonary hypertension although none of them needed extracorporeal life support. Fig 6 shows surfactant function of MAS patients during dynamic compression-expansion cycling before and during WBH, when the lowest minimum surface tension is achieved (that is, after 72h and 48h of hypothermia for patient 1 and 2, respectively). The minimum surface tension value is obtained only by analysing samples at 33.5°C (Fig 6). Cholesterol amount is mostly halved (from 52 (1.8) % before WBH to 35 (2.8) % at 72h and from 87 (3.1) % before WBH to 20 (1.3) % at 48h) for patient 1 and 2 respectively, while oxygenation and pH are improved.

**Table 4:** Basic characteristics of neonates with meconium aspiration syndrome. Both neonates were severely ill and treated with aggressive ventilatory support, surfactant broncho-alveolar lavage followed by surfactant bolus (more details in the text). PPHN was treated with inhaled nitric oxide.

	Patient 1	Patient 2
Gestational age (weeks)	41	38
Birth weight (grams)	3480	2470
SNAPPE-II score	66	58
5’ Apgar score	4	6
Type of delivery	Vaginal	C-section
Sex	Female	Female
Cord pH	6.9	7.1
OI at the NICU admission	18.5	12.5
Type of ventilation	HFOV	HFOV
Surfactant lavage and bolus (h of life)	5	3,5
Co-morbidities	PPHN	PPHN
NICU stay (d)	16	22
Outcome	Alive	Alive

Abbreviations: HFOV: high frequency oscillatory ventilation; MAS: meconium aspiration syndrome; NICU: neonatal intensive care unit; PPHN: Persistent pulmonary hypertension of the neonate; SNAPPE-II Score for Neonatal Acute Physiology-Perinatal Extension-II

Consistently, best compressibility is achieved during the measurements at 33.5°C: the slope increases from 73.8 (5.1) mN/m before WBH to 82.1 (1.9) mN/m at 72h and from 59.7 (2.1) mN/m before WBH to 83.8 (4.4) mN/m at 48h for patients 1 and 2, respectively.

## Discussion

To the best of our knowledge, scanty data are available about the effect of WBH on biophysical properties of human surfactant. In a previous preliminary study, we showed improved interfacial adsorption at 48h of hypothermia [9]. However, these data were obtained analysing the mere kinetics of surfactant accumulation into the air/liquid interface in a simplified plate fluorescent assay [33]. This technique can only assess the capability of surfactant to form a surface-associated layer, but it cannot provide any detailed information about surface tension and how surfactant stabilizes the interface during breathing-like compression-expansion cycles.

We performed here a complete CBS study and demonstrated that WBH might improve surfactant biophysical activity. Our data were provided running the experiments both at physiological and WBH temperature and mimicking the respiratory cycles with cyclic compression-expansion conditions [34]. Moreover, our findings are fully consistent with those previously obtained in rabbits using a similar study design, but with surfactant properties analysed in a Langmuir balance under conditions less comparable with actual lung mechanics than those reproduced in the CBS [35].

We observed no significant difference in adsorption during biofilm formation between each time point of WBH. The surface tension after 5’ from surfactant injection was also similar at the two temperatures. Nevertheless, at 33.5°C there was a slightly slower achievement of equilibrium. This may be explained by a different speed of material organization at the interface: the lower temperature could increase the time needed to reach the equilibrium without modifying the achieved surface tension, as already described for porcine surfactant tested at 25°C [13]. Although we did not observe relevant differences during initial adsorption, we obtained interesting results under compression-expansion cycling. From 48-72h of hypothermia onwards, surfactant showed a significantly different biophysical behavior at different temperatures: surface tension decreases only at 33.5°C. This time-dependent improvement is consistent with our previous results with the surfactant adsorption test [9]. Thus, after a given time spent under hypothermia, human surfactant shows appropriate compressibility and is able to lower surface tension along repetitive compression-expansion cycling. For surfactant film to reach and maintain low surface tensions, a multi-layered, highly cohesive structure seems to be required [36]. Our data suggest that time is needed to create such a multi-layered structure. The difference in surfactant activity between the two experimental temperatures remains significant shortly after re-warming. However, we do not know how long the effect of WBH might be maintained, as it is not possible to perform BAL in non-intubated neonates for ethical reasons. Consistently, pre-WBH sample obtained when neonates were still in normothermia did not show any improvement when tested at 33.5°C *ex vivo*.

We speculate that there are time-dependent structural reorganizations and/or compositional changes of surfactant starting from 48-72h of WBH and persisting in the first 6h after rewarming. These changes aim to improve surfactant function at 33.5°C allowing for a more efficacious gas exchange under these conditions. Similar changes have been described in hibernating animals: varying temperature leads to adaptive variations in surfactant structure and/or composition [13,14]. These temperature variations must take place within 24h and may be related to an increase of cholesterol in surfactant membranes. The steroid may be rapidly mobilized from pre-existing surfactant stores, allowing very rapid changes in material organization and adaptation to environmental conditions [13,14].

It is well known that surfactant composition sustains a coexistence of ordered and disordered phases at physiological temperatures. This duality is related to two apparently contradictory properties: the capability of rapid adsorption and re-spreading at the interface as well as the high mechanical stability of material during compression cycles [37]. It was also demonstrated that a temperature of 30°C or lower reduces the lamellar bodies particle adsorption by a decrease in the fluidity of the surfactant phases [38]. Conversely, cholesterol has a fluidizing effect on lipids bilayers in the gel state, leading to a packing-disruption of membranes [39]. When present at physiological level, its effect seems to play a critical role in promoting membrane organization, hence facilitating the spreading properties of the porcine surfactant [16]. During hypothermia, we obtained a trend of cholesterol to increase at 24-48h (of around 15–20% of the basal amount) in 5 out of 8 patients, but the steroid percentage still remains comparable with the levels described for porcine and human neonatal surfactant [16,40]. This slight increase in endogenous cholesterol precedes the improvement in biophysical activity at 72h without affecting surfactant properties. We can speculate that this process may be a physiological response to counteract the loss in fluidity or/and a regulatory mechanism to compensate environmental changes. However, this tendency is not significant due to the small population size.

Another possible explanation for the improvement in surfactant activity, is that hypothermia could stimulate the *in vivo* production or secretion of endogenous surfactant. However, DPPC pool and half-life have been reported to be unaffected by WBH [11]. Variations in other surfactant phospholipids or, alternatively, mobilization of surfactant components from pre-existing reservoirs could also play a role. Indeed, in surfactant samples from upper airways, palmitoylmyristoylphosphatidylcholine (PC 16:0/14:0) is up-regulated during human alveolar maturation [41] at the expense of DPPC. This process also explains the variations in surfactant activity among pre-term babies whose material was tested at the same total choline concentration [42]. Moreover, other phospholipids variations have been also suggested in mammals to adapt surfactant function according to environmental conditions, such as strenuous exercise [43] or body temperature fluctuation [13,14].

According to recent clinical data, WBH seems to improve oxygenation and clinical outcomes in MAS neonates [10]. Case reports also suggested a beneficial effect in older patients with ARDS [4,5]. These possible benefits may be partially explained by our findings. In MAS patients, surface tension under dynamic cycling reaches the lowest value after 48-72h of hypothermia, while surfactant compressibility is at its highest at the same time. This means that a minimal compression is sufficient for a rapid decrease in surface tension. Consistently, these results were not confirmed when running the experiment at 37°C or in BAL samples obtained before the WBH instigation. Surfactant cholesterol was very much reduced when the minimum surface tension was reached in MAS patients: although physiological amount of cholesterol helps surfactant to assume its correct structure, an excess of cholesterol (i.e. in the order of 20% or above with respect to the PL concentration) is known to impair surfactant capability to reach low surface tension under compression-expansion conditions [44–47]. This is consistent with the fact that the addition of DPPC stabilizes the membranes properties and restores surfactant activity [47]. As previously described [47], we observed a very high level of cholesterol in samples of MAS neonates (52 and 87% with respect to total PC choline, equivalent to around 17 and 30% with respect to phospholipids). The amount was 60–80% higher than that obtained in NLD babies. Moreover, surfactant activity in MAS neonates was impaired during pre-hypothermia and improved only when a cholesterol reduction emerges during cooling (from 48h onward). In this context, the enhancement in surface tension was evident only when surfactant was tested at 33.5°C.

We can speculate on two intriguing aspects. First, the dual role of cholesterol. The slight increase of the steroid may modulate physiological responses to environmental conditions without damaging surfactant functionality, whereas its excess and the consequent high fluidity may impair the capability of material to achieve minimal surface tensions. Second, the different phospholipid re-organization at 33.5°C. The temperature reduction might cause a selective exclusion of low-compressible lipids (such as different unsaturated phospholipids mobilized at 48-72h of WBH) from the alveolar interface to obtain a DPPC-enriched film with a solid-ordered type of phase during expiration. This process may be evident upon testing surfactant by *in vitro* breathing-like compression-expansion cycles, although the *in vivo* metabolic production of DPPC does not change.

We observed an improvement in oxygenation and pH in MAS patients, when minimum surface tension was achieved. Beside these findings, we cannot exclude other physiopathological mechanisms such as: 1) WBH-induced reduction in metabolic demand and CO_2_ production with consequent need for a less aggressive ventilation [2,48], 2) the effect of surfactant lavage and replacement [38,49] and 3) the reduction in lung inflammation and proteins extravasation by WBH [8,9,50–52]. This hypothesis does not exclude the possible variations in PC molecular species during hypothermia. Some reports have already demonstrated that the increase in PC16:0/14:0 is related to the inhibition of macrophage-triggered proliferation of T-lymphocytes and oxygen radicals production [53,54].

We acknowledge some study limitations. First, there is no control group of babies with repeated BAL under normothermia. This group is impossible to be recruited because it is unethical to keep intubated healthy neonates for 72h only to perform BALs; conversely, neonates may stay intubated so long if they have lung diseases or perinatal asphyxia needing WBH, and thus they cannot represent healthy controls. Our measurements pre-WBH must be considered as control group. Indeed, although the surfactant does not reach near-zero surface tension at the dynamic experiments, the same performance was already described for healthy babies in literature by using a similar pre-assay treatment [55,9]. This sub-optimal performance is mainly related to the following critical points: 1) the material is only precipitated and not purified as it happens in animal studies; 2) we obtained very low concentration from non-bronchoscopic BAL samples, which makes impossible to perform experiments at phospholipid concentrations in the order of 20–25 mg/mL, as usually applied to test porcine surfactant [56]. On the other hand, the use of a limiting concentration of 8 mg/mL in the functional tests allows the detection of subtle differences in the biophysical parameters at different temperatures. Second, we enrolled a small sample size, although similar to other studies in the field [9,43]: this is due to the rarity of these patients, especially those affected by MAS. Larger populations could ideally be enrolled with multicenter studies but a complete CBS study is a very complex and time-consuming procedure, thus such studies should run for several years and are not realistic. Third, we did not perform a fibroscopic BAL and instead we used the non-bronchoscopic BAL, as commonly performed in neonates [26]. The non-bronchoscopic technique might affect results, as it may harvest surfactant also from the upper airways and not only from the alveolar *milieu*. The low surfactant concentration of tested samples did not allow us to perform a detailed surfactant protein quantification (by SDS gel or ELISA test) and better characterize the quality of material. However, D9-choline labeling of healthy volunteers showed that upper airways samples still reflect alveolar PC composition and metabolism [41]. The fibroscopic technique is not suitable in neonates due to the small patients’ size and studies on neonatal lung biology are commonly done with the non-bronchoscopic BAL [9,11,25,28]. Moreover, our technique for non-bronchoscopic BAL is standardized and is known to describe the alveolar *milieu* better than the simple tracheal aspirates [57]. A total lung lavage would have also retrieved a surfactant of better quality, as done in animal studies [13,14], but this technique is not applicable to human patients for ethical reasons. Moreover, NLD babies cannot be considered as a perfect control group of healthy neonates, since they are anyway affected by perinatal asphyxia. Ideally, normal surfactant should be studied in spontaneously breathing neonates, as there is the theoretical risk that mechanical ventilation, even if provided according to the best current clinical practice, might affect surfactant quality/function. However, performing a BAL on non-intubated babies in unethical and unfeasible, thus NLD is the best control available for this type of study. Finally, a lipidomic and differential scanning calorimetry thermal analysis would have clarified if there are other surfactant compositional and structural changes beyond cholesterol concentration: unfortunately, neonatal BAL samples consist of a very low volume which was entirely needed for the biophysical study. This issue will be addressed with a specific future study.

In conclusion, surfactant biophysical properties improve after at least 48-72h of WBH in human neonates with or without MAS and the improvement is maintained shortly after rewarming. The improvement seems related to the WBH–induced surfactant compositional/structural changes. These findings, together with the other local and systemic effects of hypothermia, may partially explain the clinical benefit provided by WBH in some cases of respiratory failure.

## References

[1] Committee on Fetus and Newborn, Papile LA, Baley JE, Benitz W, Cummings J, Carlo WA, et al Hypothermia and neonatal encephalopathy. Pediatrics 2014;133:1146–50. doi: 10.1542/peds.2014-0899 24864176 10.1542/peds.2014-0899

[2] Pietrini D, Piastra M, Luca E, Mancino A, Conti G, Cavaliere F, et al Neuroprotection and hypothermia in infants and children. Curr Drug Targets 2012;13:925–35 22512392 10.2174/138945012800675641

[3] Jacobs SE, Berg M, Hunt R, Tarnow-Mordi WO, Inder TE, Davis PG. Cooling for newborns with hypoxic ischaemic encephalopathy. Cochrane Database Syst Rev 2013;1:CD003311. 10.1002/14651858.CD003311.pub3 PMC7003568 23440789

[4] Pietrini D, Pennisi M, Vitale F, Pulitanò SM, Conti G, Mancino A, et al Rescue hypothermia for refractory hypercapnia. Eur J Pediatr 2012;171:1855–7 doi: 10.1007/s00431-012-1769-6 22692802 10.1007/s00431-012-1769-6

[5] Duan M, Berra L, Kumar A, Wilcox S, Safford S, Goulet R, et al Use of hypothermia to allow low-tidal-volume ventilation in a patient with ARDS. Respir Care 2011;56:1956–8. doi: 10.4187/respcare.01211 21682985 10.4187/respcare.01211

[6] Dhillon G, Gopal PB, Kamat AS, Mulavisala KP. Induced hypothermia for trauma-related ARDS. Indian J Crit Care Med 2015;19:353–5. doi: 10.4103/0972-5229.158278 26195862 10.4103/0972-5229.158278 PMC4478677

[7] Hall NJ, Eaton S, Peters MJ, Hiorns MP, Alexander N, Azzopardi DV, et al Mild controlled hypothermia in preterm neonates with advanced necrotizing enterocolitis. Pediatrics 2010;125:e300–8. doi: 10.1542/peds.2008-3211 20100756 10.1542/peds.2008-3211

[8] Ball MK, Hillman NH, Kallapur SG, Polglase GR, Jobe AH, Pillow JJ. Body temperature effects on lung injury in ventilated preterm lambs. Resuscitation 2010;81:749–54. doi: 10.1016/j.resuscitation.2009.12.007 20299144 10.1016/j.resuscitation.2009.12.007 PMC2871967

[9] De Luca D, Vázquez-Sánchez S, Minucci A, Echaide M, Piastra M, Conti G, et al Effect of whole body hypothermia on inflammation and surfactant function in asphyxiated neonates. Eur Respir J 2014;44:1708–10. doi: 10.1183/09031936.00117714 25142478 10.1183/09031936.00117714

[10] De Luca D, Tingay DG, van Kaam A, Brunow de Carvalho W, Valverde E, Christoph Roehr C, et al IGLOO Study Group. Hypothermia and Meconium Aspiration Syndrome: International Multicenter Retrospective Cohort Study. Am J Respir Crit Care Med 2016;194:381–4. doi: 10.1164/rccm.201602-0422LE 27479063 10.1164/rccm.201602-0422LE

[11] Nespeca M, Giorgetti C, Nobile S, Ferrini I, Simonato M, Verlato G, et al Does Whole-Body Hypothermia in Neonates with Hypoxic-Ischemic Encephalopathy Affect Surfactant Disaturated-Phosphatidylcholine Kinetics? Plos One 2016;11:e0153328 doi: 10.1371/journal.pone.0153328 27070307 10.1371/journal.pone.0153328 PMC4829158

[12] Perez-Gil J. Structure of pulmonary surfactant membranes and films: the role of proteins and lipid-protein interactions. Biochim Biophys Acta 2008; 1778:1676–95. doi: 10.1016/j.bbamem.2008.05.003 18515069 10.1016/j.bbamem.2008.05.003

[13] Suri LN, McCaig L, Picardi MV, Ospina OL, Veldhuizen RA, Staples JF, et al Adaptation to low body temperature influences pulmonary surfactant composition thereby increasing fluidity while maintaining appropriately ordered membrane structure and surface activity. Biochim Biophys Acta 2012;1818:1581–9. doi: 10.1016/j.bbamem.2012.02.021 22387458 10.1016/j.bbamem.2012.02.021

[14] Suri LN, Cruz A, Veldhuizen RA, Staples JF, Possmayer F, Orgeig S, et al Adaptations to hibernation in lung surfactant composition of 13-lined ground squirrels influence surfactant lipid phase segregation properties. Biochim Biophys Acta 2013;1828:1707–14. doi: 10.1016/j.bbamem.2013.03.005 23506681 10.1016/j.bbamem.2013.03.005

[15] Parra E, Pérez-Gil J. Composition, structure and mechanical properties define performance of pulmonary surfactant membranes and films. Chem Phys Lipids. 2015;185:153–75. doi: 10.1016/j.chemphyslip.2014.09.002 25260665 10.1016/j.chemphyslip.2014.09.002

[16] Bernardino de la Serna J, Perez-Gil J, Simonsen AC, Bagatolli LA. Cholesterol rules: direct observation of the coexistence of two fluid phases in native pulmonary surfactant membranes at physiological temperatures. J Biol Chem. 2004;279:40715–22. doi: 10.1074/jbc.M404648200 15231828 10.1074/jbc.M404648200

[17] Yu SH, Possmayer F. Adsorption, compression and stability of surface films from natural, lipid extract and reconstituted pulmonary surfactants. Biochim Biophys Acta 1993;1167:264–271. 8481387 10.1016/0005-2760(93)90228-2

[18] Notter RH, Tabak SA, Mavis RD. Surface properties of binary mixtures of some pulmonary surfactant components. J Lipid Res. 1980;21(1):10–22. 6892572

[19] Hawco MW, Davis PJ, Keough KM. Lipid fluidity in lung surfactant: monolayers of saturated and unsaturated lecithins. J Appl Physiol 1981;51:509–515. doi: 10.1152/jappl.1981.51.2.509 6894918 10.1152/jappl.1981.51.2.509

[20] Bernardino de la Serna J, Orädd G, Bagatolli LA, Simonsen AC, Marsh D, Lindblom G et al Segregated phases in pulmonary surfactant membranes do not show coexistence of lipid populations with differentiated dynamic properties. Biophys J 2009;97(5):1381–9. doi: 10.1016/j.bpj.2009.06.040 19720026 10.1016/j.bpj.2009.06.040 PMC2749771

[21] Autilio C, Echaide M, Dell’Orto V, Perez-Gil J, De Luca D. Effect of Whole Body Hypothermia on Surfactant Function When Amniotic Fluid is Meconium Stained. Ther Hypothermia Temp Manag 2017; forthcoming. doi: 10.1089/ther.2017.0012 28708464 10.1089/ther.2017.0012

[22] Azzopardi DV, Strohm B, Edwards AD, Dyet L, Halliday HL, Juszczak E, et al TOBY Study Group. Moderate hypothermia to treat perinatal asphyxial encephalopathy. N Engl J Med 2009;361:1349–58. doi: 10.1056/NEJMoa0900854 19797281 10.1056/NEJMoa0900854

[23] De Jaegere A, van Veenendaal MB, Michiels A, van Kaam AH. Lung recruitment using oxygenation during open lung high-frequency ventilation in preterm infants. Am J Respir Crit Care Med 2006;174:639–45. doi: 10.1164/rccm.200603-351OC 16763218 10.1164/rccm.200603-351OC

[24] Boynton BR, Hammond MD, Fredberg JJ, Buckley BG, Villanueva D, Frantz ID. Gas exchange in healthy rabbits during high frequency oscillatory ventilation. J Appl Physiol 1989;66:1343–1351. doi: 10.1152/jappl.1989.66.3.1343 2496093 10.1152/jappl.1989.66.3.1343

[25] Piastra M, Yousef N, Brat R, Manzoni P, Mokhtari M, De Luca D. Lung ultrasound findings in meconium aspiration syndrome. Early Hum Dev 2014;90 Suppl 2:S41–3 25220126 10.1016/S0378-3782(14)50011-4

[26] Dargaville PA, Copnell B, Mills JF, Haron I, Lee JK, Tingay DG, et al Randomized controlled trial of lung lavage with dilute surfactant for meconium aspiration syndrome. J Pediatr 2011;158:383–389.e2 doi: 10.1016/j.jpeds.2010.08.044 20947097 10.1016/j.jpeds.2010.08.044

[27] De Luca D, Minucci A, Tripodi D, Piastra M, Pietrini D, Zuppi C, et al Role of distinct phospholipases A2 and their modulators in meconium aspiration syndrome in human neonates. Intensive Care Med 2011;37:1158–65. doi: 10.1007/s00134-011-2243-z 21567110 10.1007/s00134-011-2243-z

[28] de Blic J, Midulla F, Barbato A, Clement A, Dab I, Eber E, et al Bronchoalveolar lavage in children. ERS Task Force on bronchoalveolar lavage in children. Eur Respir J 2000;15:217–231. 10678650 10.1183/09031936.00.15121700

[29] Nanjee MN, Gebre AK, Miller NE. Enzymatic fluorometric procedure for phospholipid quantification with an automated microtiter plate fluorometer. Clin Chem 1991;37:868–74. 1904801

[30] Naito HK, David JA. Laboratory considerations: determination of cholesterol, triglycerides, phospholipid and others lipids in blood and tissues. Lab Res Methods Biol Med 1984;10:1–76. 6390047

[31] Schürch S, Green FH, Bachofen H. Formation and structure of surface films: captive bubble surfactometry. Biochim Biophys Acta 1998;1408:180–202. 9813315 10.1016/s0925-4439(98)00067-2

[32] Schoel WM, Schürch S, Goerke J. The captive bubble method for the evaluation of pulmonary surfactant: surface tension, area, and volume calculations. Biochim Biophys Acta 1994;1200:281–90. 8068714 10.1016/0304-4165(94)90169-4

[33] Ravasio A, Cruz A, Perez-Gil J, Haller T. High-throughput evaluation of pulmonary surfactant adsorption and surface film formation. J Lipid Res 2008;49:2479–88. doi: 10.1194/jlr.D800029-JLR200 18641374 10.1194/jlr.D800029-JLR200

[34] Serrano AG and Perez-Gil J. Protein-lipid interactions and surface activity in the pulmonary surfactant system. Chem Phys Lipids 2006;141:105–118. doi: 10.1016/j.chemphyslip.2006.02.017 16600200 10.1016/j.chemphyslip.2006.02.017

[35] Lempert J, Macklem PT. Effect of temperature on rabbit lung surfactant and pressure-volume hysteresis. J Appl Physiol 1971;31:380–5 doi: 10.1152/jappl.1971.31.3.380 5170908 10.1152/jappl.1971.31.3.380

[36] Bernardino de la Serna J, Vargas R, Picardi V, Cruz A, Arranz R, Valpuesta JM, et al Segregated ordered lipid phases and protein-promoted membrane cohesivity are required for pulmonary surfactant films to stabilize and protect the respiratory surface. Faraday Discuss 2013;161:535–48. 23805757 10.1039/c2fd20096a

[37] Casals O. Canadas. Role of lipid ordered/disordered phase coexistence in pulmonary surfactant function. Biochim Biophys Acta 2012; 1818:2550–62. doi: 10.1016/j.bbamem.2012.05.024 22659676 10.1016/j.bbamem.2012.05.024

[38] Hobi N, Siber G, Bouzas V, Ravasio A, Pérez-Gil J, Haller T. Physiological variables affecting surface film formation by native lamellar body-like pulmonary surfactant particles. Biochim Biophys Acta 2014;1838:1842–50. doi: 10.1016/j.bbamem.2014.02.015 24582711 10.1016/j.bbamem.2014.02.015

[39] Lopez-Rodriguez E, Pérez-Gil J. Structure-function relationships in pulmonary surfactant membranes: from biophysics to therapy. Biochim Biophys Acta 2014;1838:1568–85. doi: 10.1016/j.bbamem.2014.01.028 24525076 10.1016/j.bbamem.2014.01.028

[40] Griese M, Kirmeier HG, Liebisch G, Rauch D, Stückler F, Schmitz G et al Surfactant lipidomics in healthy children and childhood interstitial lung disease. PLoS One 2015;10:e0117985 doi: 10.1371/journal.pone.0117985 25692779 10.1371/journal.pone.0117985 PMC4333572

[41] Bernhard W. Lung surfactant: Function and composition in the context of development and res-piratory physiology. Ann Anat. 2016;208:146–150. doi: 10.1016/j.aanat.2016.08.003 27693601 10.1016/j.aanat.2016.08.003

[42] Autilio C, Echaide M, Benachi A, Marfaing-Koka A, Capoluongo ED, Pérez-Gil J. A Noninvasive Surfactant Adsorption Test Predicting the Need for Surfactant Therapy in Preterm Infants Treated with Continuous Positive Airway Pressure. J Pediatr. 2017;182:66–73.e1. doi: 10.1016/j.jpeds.2016.11.057 27989413 10.1016/j.jpeds.2016.11.057

[43] Doyle IR, Jones ME, Barr HA, Orgeig S, Crockett AJ, McDonald CF, et al Composition of human pulmonary surfactant varies with exercise and level of fitness. Am J Respir Crit Care Med 1994;149:1619–27. doi: 10.1164/ajrccm.149.6.8004321 8004321 10.1164/ajrccm.149.6.8004321

[44] Gunasekara L, Schürch S, Schoel WM, Nag K, Leonenko Z, Haufs M, et al Pulmonary surfactant function is abolished by an elevated proportion of cholesterol. Biochim Biophys Acta 2005;1737:27–35. doi: 10.1016/j.bbalip.2005.09.002 16216549 10.1016/j.bbalip.2005.09.002

[45] Keating E, Rahman L, Francis J, Petersen A, Possmayer F, Veldhuizen R, et al Effect of cholesterol on the biophysical and physiological properties of a clinical pulmonary surfactant. Biophys J 2007;93:1391–401. doi: 10.1529/biophysj.106.099762 17526587 10.1529/biophysj.106.099762 PMC1929052

[46] Keating E, Rahman L, Francis J, Petersen A, Possmayer F, Veldhuizen R, et al Effect of cholesterol on the biophysical and physiological properties of a clinical pulmonary surfactant. Biophys J. 2007 8 15;93(4):1391–401. doi: 10.1529/biophysj.106.099762 17526587 10.1529/biophysj.106.099762 PMC1929052

[47] Echaide M, Autilio C, Arroyo R, Perez-Gil J. Restoring pulmonary surfactant membranes and films at the respiratory surface. Biochim Biophys Acta 2017;1859:1725–1739. doi: 10.1016/j.bbamem.2017.03.015 28341439 10.1016/j.bbamem.2017.03.015

[48] Aslami H, Binnekade JM, Horn J, Huissoon S, Juffermans NP. The effect of induced hypothermia on respiratory parameters in mechanically ventilated patients. Resuscitation 2010;81:1723–5. doi: 10.1016/j.resuscitation.2010.09.006 20947237 10.1016/j.resuscitation.2010.09.006

[49] Boet A, Brat R, Aguilera SS, Tissieres P, De Luca D. Surfactant from neonatal to pediatric ICU: bench and bedside evidence. Minerva Anestesiol 2014;80:1345–56. 24504167

[50] Altınsoy C, Tuzun F, Duman N, Sever AH, Dilek M, Ozbal S, et al Effect of induced hypothermia on lipopolysaccharide-induced lung injury in neonatal rats. J Matern Fetal Neonatal Med 2014;27:421–9. doi: 10.3109/14767058.2013.818115 23795616 10.3109/14767058.2013.818115

[51] Hong SB, Koh Y, Lee IC, Kim MJ, Kim WS, Kim DS, et al Induced hypothermia as a new approach to lung rest for the acutely injured lung. Crit Care Med 2005;33:2049–55. 16148479 10.1097/01.ccm.0000178186.37167.53

[52] Aslami H, Kuipers MT, Beurskens CJ, Roelofs JJ, Schultz MJ, Juffermans NP. Mild hypothermia reduces ventilator-induced lung injury, irrespective of reducing respiratory rate. Transl Res 2012;159:110–7. doi: 10.1016/j.trsl.2011.10.005 22243795 10.1016/j.trsl.2011.10.005

[53] Gille C, Spring B, Bernhard W, Gebhard C, Basile D, Lauber K, et al Differential effect of surfactant and its saturated phosphatidylcholines on human blood macrophages. J Lipid Res 2007;48:307–317. doi: 10.1194/jlr.M600451-JLR200 17099186 10.1194/jlr.M600451-JLR200

[54] Bernhard W, Raith M, Pynn CJ, Gille C, Stichtenoth G, Stoll D, et al Increased palmitoyl-myristoyl-phosphatidylcholine in neonatal rat surfactant is lung specific and correlates with oral myristic acid supply. J Appl Physiol 2011;111:449–457. doi: 10.1152/japplphysiol.00766.2010 21636561 10.1152/japplphysiol.00766.2010

[55] De Luca D, Lopez-Rodriguez E, Minucci A, Vendittelli F, Gentile L, Stival E et al Clinical and biological role of secretory phospholipase A2 in acute respiratory distress syndrome infants. Crit Care 2013;17:R163 doi: 10.1186/cc12842 23883784 10.1186/cc12842 PMC4057254

[56] Lopez-Rodriguez E, Echaide M, Cruz A, Taeusch HW, Perez-Gil J. Meconium impairs pulmonary surfactant by a combined action of cholesterol and bile acids. Biophys J 2011;100:646–55. doi: 10.1016/j.bpj.2010.12.3715 21281579 10.1016/j.bpj.2010.12.3715 PMC3030210

[57] Dargaville PA, South M, McDougall PN. Comparison of two methods of diagnostic lung lavage in ventilated infants with lung disease. Am J Respir Crit Care Med 1999;160:771–7. doi: 10.1164/ajrccm.160.3.9811048 10471595 10.1164/ajrccm.160.3.9811048

